# Association between perirenal fat tissue thickness and subclinical atherosclerosis in patients newly diagnosed with type 2 diabetes: a cross-sectional study

**DOI:** 10.12701/jyms.2026.43.10

**Published:** 2026-01-06

**Authors:** Işıl Isel, Mehmet Karagulle, Turgut Karabag

**Affiliations:** 1 Department of Internal Medicine, Istanbul Education and Research Hospital, Saglik Bilimleri University, Istanbul, Türkiye; 2 Department of Radiology, Istanbul Education and Research Hospital, Saglik Bilimleri University, Istanbul, Türkiye; 3 Department of Cardiology, Istanbul Education and Research Hospital, Saglik Bilimleri University, Istanbul, Türkiye

**Keywords:** Atherosclerosis, Intraabdominal fat, Type 2 diabetes mellitus, Ventricular dysfunction

## Abstract

**Background:**

Perirenal fat is an important endocrine organ that produces and secretes bioactive cytokines and adipokines involved in the pathogenesis of cardiovascular diseases. The association of perirenal and pararenal fat tissue thickness (PPRFT) with subclinical atherosclerosis and myocardial function in patients with newly diagnosed type 2 diabetes mellitus (ND-T2DM) was investigated in this study.

**Methods:**

The study included 111 patients with ND-T2DM (59 males; mean age, 49.7±9.6 years) and 57 individuals without any disease diagnosis as the control group (23 males; mean age, 48.5±7.2 years). PPRFT and carotid intima-media thickness (CIMT) were measured using ultrasonography. Conventional parameters, including epicardial fat tissue (EFT) thickness, were measured using transthoracic echocardiography and myocardial velocities were measured using tissue Doppler echocardiography.

**Results:**

CIMT, EFT thickness, and PPRFT were higher in patients with ND-T2DM than in controls (*p*<0.001). While the E/A ratio was significantly lower in patients with ND-T2DM than in controls (*p*<0.001), the E wave deceleration time, and E/E′ septal and lateral ratios were significantly higher in the former (*p*<0.001, *p*<0.001, and *p*=0.002, respectively). PPRFT values were significantly correlated with CIMT and EFT thickness (ρ=0.490, ρ=0.517; *p*<0.001 and ρ=0.588, ρ=0.574; *p*<0.001, respectively) and negatively correlated with ejection fraction and mitral E/A ratio (ρ=–0.549, ρ=–0.530; *p*<0.001 and ρ=–0.512, ρ=–0.465; *p*<0.001, respectively).

**Conclusion:**

PPRFT values measured using ultrasonography in patients with ND-T2DM are significantly associated with CIMT and EFT, which are indicators of subclinical atherosclerosis.

## Introduction

Diabetes mellitus (DM) is a condition in which chronic hyperglycemia occurs because of a defect in the production or action of insulin [1]. Type 2 diabetes mellitus (T2DM), also known as adult-onset DM, is the most common type of DM and accounts for the majority of DM cases in the world [2]. T2DM affects multiple systems and is associated with initial microvascular and progressive macrovascular complications [3]. Adiposity is associated with various cardiovascular diseases including hypertension and DM. In addition to general body fat, different types of fat accumulation are associated with various cardiovascular risks [4]. Visceral adipose tissue plays a greater role in the pathogenesis of T2DM than subcutaneous adipose tissue [5]. Perirenal adipose tissue (PAT) is an important component of visceral adipose tissue and is localized in the retroperitoneal region surrounding the kidneys. Emerging evidence has highlighted that PAT may functionally regulate the cardiovascular system through several mechanisms such as adipokine secretion, adipocyte interactions, and paracrine signaling. Therefore, PAT may be a potential target for preventing cardiovascular disease and atherosclerosis [6-8].

Subclinical atherosclerosis is the presence of atheromatous disease in one or more arterial territories before the appearance of any signs, symptoms, or events attributable to the resulting atherosclerotic disease. Early detection of subclinical atherosclerosis is important because timely intervention and precautions can prevent future cardiovascular diseases [9].

In this study, we investigated the relationship between perirenal and pararenal fat tissue thickness (PPRFT), measured using ultrasonography, and subclinical atherosclerosis in patients with newly diagnosed T2DM (ND-T2DM). The relationship between PPRFT and laboratory findings as well as myocardial functions obtained by echocardiography was also investigated.

## Methods

**Ethics statement:** This study was approved by the Local Ethics Committee of Sağlık Bilimleri Üniversitesi, Istanbul Education and Research Hospital (approval number: 397). This study was conducted in accordance with the ethical standards specified in the 1964 Declaration of Helsinki and its later amendments or comparable ethical standards.

### 1. Patients

This was a cross-sectional study that included 168 consecutive patients. One hundred and eleven patients with ND-T2DM and 57 patients without DM, who were over 18 years of age, had provided study consent, and were sufficiently cooperative and orientated, were included in the study (86 females and 82 males; mean age, 49.3±8.9 years). Patients with preexisting type 1 DM; coronary artery disease; chronic liver and kidney disease; severe chronic diseases such as malignancy; additional metabolic disease; active infection; history of antidiabetic, antihypertensive, or lipid-lowering drug use; and patients who were pregnant were excluded from the study. Individuals taking medications that directly or indirectly affect cardiac function, heart rate, or blood pressure were also excluded.

Detailed medical histories were obtained from the patients, and physical examinations were performed for those who met the inclusion criteria. Systolic and diastolic blood pressure, heart rate, body weight (kg), height (cm), and hip and waist circumferences in centimeters were measured and recorded. Hip and waist circumferences were measured with a tape measure. Specifically, the patient was in a standing position with arms open to the sides; waist circumference was measured by combining the umbilicus level in front and the subcostal region on the sides, and hip circumference was measured by combining the symphysis pubis in front and the most protruding part of the gluteal region in the back.

T2DM was diagnosed according to the American Diabetes Association criteria. The diagnostic thresholds were as follows: a fasting plasma glucose level of ≥126 mg/dL (7.0 mmol/L), a 2-hour plasma glucose level of ≥200 mg/dL (11.1 mmol/L) during a 75-g oral glucose tolerance test, a random plasma glucose level of ≥200 mg/dL (11.1 mmol/L) in a patient with classic symptoms of hyperglycemia or hyperglycemic crisis, or a glycated hemoglobin (HbA1c) level of ≥6.5% (48 mmol/mol) [10].

### 2. Biochemical examinations

Blood and urine samples were collected from all participants at the same time in the morning after an overnight fast of ≥8 hours. Blood glucose, urea, creatinine, aspartate aminotransferase, alanine aminotransferase (ALT), HbA1c, gamma-glutamyl transferase, total cholesterol, low-density lipoprotein cholesterol, triglyceride, high-density lipoprotein cholesterol (HDL-C), uric acid, C-reactive protein (CRP), insulin, hemogram, and urine microalbumin levels were measured and recorded.

### 3. Echocardiographic imaging and carotid intima-media thickness measurements

Transthoracic echocardiography was performed on patients in the left lateral decubitus position using a Philips EPIQ 7 echocardiography device (Philips Healthcare, Andover, MA, USA) with a 2.5 to 3.5 MHz transducer. M-mode recordings were performed at 50 mm/second, and Doppler recordings were performed at 100 mm/second. All the measurements were performed under electrocardiographic guidance. M-mode and two-dimensional (2D) measurements were obtained from the parasternal long-axis view. Conventional echocardiographic parameters (left atrium and ventricle dimensions and wall thickness), Doppler diastolic parameters (mitral E, A waves, E wave deceleration time [EDT], and mitral E/A), and tissue Doppler parameters measured from the lateral, septal, and tricuspid annuli (S′, E′, A′) were obtained. Ejection fraction (EF) was calculated using the Simpson method. Diastolic functions were evaluated with diastolic velocities measured from septal and lateral regions (E/E′ septal, E/E′ lateral, respectively). Epicardial fat tissue thickness was measured from at least two locations on the right ventricular free wall from both the parasternal long axis and transverse parasternal views. Carotid intima-media thickness (CIMT) was measured 2 to 3 cm above the carotid bifurcation separation in B mode with a linear transducer. All measurements were performed by the same sonographer to avoid interobserver differences, and the average of three separate measurements was recorded to reduce intraobserver differences.

### 4. Ultrasonographic measurements

**Fig. 1.**
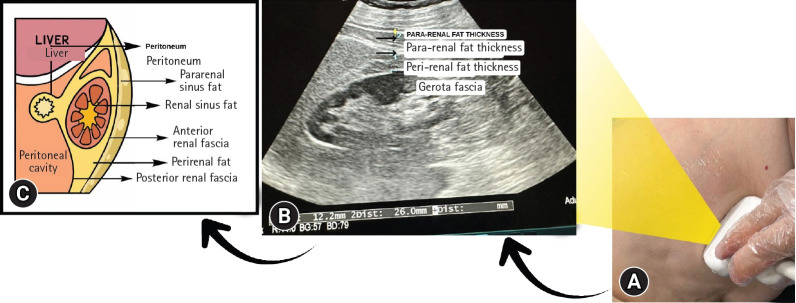
Ultrasonographic assessment of perirenal and pararenal fat thickness. (A) Placement of the ultrasound probe over the flank. (B) Ultrasonographic image showing the measurements of the perirenal and pararenal fat thicknesses. (C) A schematic diagram of retroperitoneal anatomy.

All patients included in our study were examined by the same radiologist using a high-resolution ultrasonography device (Aplio 500; Canon Medical Systems, Otawara, Japan) and a 1 to 6 MHz convex probe. Measurements of the right and left kidneys were performed separately while the patients were lying in the supine position. As a measurement standard, the convex probe was positioned on the flank region perpendicular to the transverse abdominal axis and adjusted until the longest longitudinal axis of the kidney was visualized. During the examination, the participants were instructed to take a deep breath and hold it. The maximal perirenal fat thickness was measured in this plane as the perpendicular distance between Gerota’s fascia and the renal capsule. Subsequently, total fat thickness (perirenal+pararenal) was measured as the maximal perpendicular distance between the inner layer of the abdominal wall and the renal capsule (Fig. 1). Patients with malrotation anomalies, kidneys located outside the normal location, renal masses, pelvicalyceal ectasia, or congenital renal anomalies were excluded from the study. To ensure standardization, the averages of the right and left PPRFTs were calculated. All measurements were performed using the same sonographer. The average of three separate measurements was calculated.

### 5. Statistical analysis

All data were recorded using IBM SPSS ver. 25 (IBM Corp., Armonk, NY, USA). Normal distribution of the variables was examined using histogram graphics and the Kolmogorov–Smirnov test. The mean, standard deviation, and median were used for descriptive analyses. To compare continuous variables between groups, the Student *t*-test was used if the distribution was normal, and the Mann–Whitney *U* test was used if the distribution was non-normal. Spearman correlation tests were used for the comparative analysis of the measurement data. Parameters predicting DM were included in the univariate binary logistic regression analysis, and those found to be significant were subsequently evaluated using multivariate analysis. Odds ratios (ORs) and 95% confidence intervals (CIs) were calculated. Results with a *p*-value <0.05 were considered statistically significant.

## Results

Two separate study groups were analyzed: patients with ND-T2DM (Group 1: 111 patients [59 males and 52 females]; mean age, 49.7±9.6 years) and the control group (Group 2: 57 participants [23 males and 34 females]; mean age, 48.5±7.2 years). There were no significant differences between the groups in terms of sex and smoking status. Glucose, triglyceride, CRP, and ALT levels were significantly higher in Group 1 than in Group 2. The HDL-C level was significantly lower in Group 1 than in Group 2 (Table 1). Insulin and homeostatic model assessment of insulin resistance (HOMA-IR) values were also significantly higher in Group 1 than in Group 2.

When CIMT, epicardial fat tissue thickness, and mean PPRFT values were compared between the groups, all values were significantly higher in Group 1 than in Group 2 (Table 1).

When echocardiographic findings were compared, the interventricular septum and posterior wall thicknesses and the left atrium diameter were significantly higher in Group 1 than in Group 2. Septal, lateral, and tricuspid E′ velocities among the tissue Doppler findings were significantly lower, whereas A′ velocities were significantly higher, in Group 1 than in Group 2 (Table 2). In terms of diastolic functions, while the E/A ratio was significantly lower in Group 1 than in Group 2, EDT, E/E′ septal and E/E′ lateral ratios were significantly higher (Table 2).

In the Spearman correlation test, the mean PPRFT values were significantly correlated with the epicardial fat tissue thickness (Table 3). In addition, the PPRFT values showed a significant negative correlation with the E/A ratio and EF, and a positive correlation with E/E′ septal thickness (Table 3). Similarly, there was a significant correlation with HbA1c and HOMA-IR values (Table 3). There was a moderate correlation between the mean PPRFT value and systolic and diastolic blood pressures (ρ=0.321, ρ=0.429; *p*<0.001 and ρ=0.315, ρ=0.336; *p*<0.001, respectively).

In univariate logistic regression analysis, several variables (mean pararenal thickness, mean perirenal thickness, body mass index [BMI], waist circumference, triglycerides, and HDL-C level) were significantly associated with the presence of DM. Specifically, an increase in mean pararenal thickness was associated with higher odds of DM (OR, 1.590; 95% CI, 1.369–1.848; *p*<0.001). Similarly, a higher mean perirenal thickness was significantly associated with DM (OR, 1.668; 95% CI, 1.420–1.960; *p*<0.001). After adjusting for potential confounders in the multivariate logistic regression model, both the mean pararenal thickness and the mean perirenal thickness remained independent predictors of DM. Mean pararenal thickness was associated with 1.30-fold increased odds of DM (OR, 1.296; 95% CI, 1.088–1.543; *p*=0.004), whereas mean perirenal thickness was associated with 1.36-fold increased odds (OR, 1.355; 95% CI, 1.117–1.644; *p*=0.002) (Table 4).

## Discussion

According to the results of our study, PPRFT values measured using ultrasonography in patients with ND-T2DM were significantly thicker than those in individuals without DM and were significantly associated with CIMT, an indicator of subclinical atherosclerosis. Noninvasively measured PPRFT values are related to glycemic parameters and deterioration of diastolic function, which may indicate subclinical cardiac involvement. We believe that PPRFT can be used to monitor cardiovascular involvement and glycemic parameters in these patients.

Preventing the development of atherosclerotic cardiovascular disease in individuals with DM is an important clinical imperative that should be prioritized to improve quality of life, reduce premature death, and reduce the occurrence of comorbidities, thus reducing the economic costs associated with DM [11]. Visceral adipose tissue is a type of fat that is believed to have adverse effects on various metabolic profiles, systemic inflammation, and insulin resistance, ultimately increasing the risk of developing cardiovascular disease [12,13]. Patients with T2DM have increased fat accumulation in internal organs, which enhances the risk of stroke, cardiovascular diseases, metabolic disorders, and comorbid diseases [14]. Visceral adipose tissue is the most important tissue in adipose biology considering its anatomical and physiological properties. Compared with other fatty tissues, perirenal fat tissue is known to have a complete blood supply, lymph fluid drainage, innervation, and other special morphological features that make it similar to other internal organs but different from traditionally classified connective tissues [15]. Perirenal fat may regulate the cardiovascular system through neural reflexes, adipokine secretion, adipocyte interactions, and paracrine signaling [6,7]. Adipokines contribute to lipolysis via various autocrine-, paracrine-, and endocrine-mediated pathways [16,17]. In addition, PAT is effective in stimulating the synthesis of various cytokines from local immune cells such as T lymphocytes and monocytes/macrophages, which may play an important role in triggering the inflammatory response in the endothelium [18].

Considering this information, various studies have been conducted to investigate the relationship between visceral fat tissue and cardiovascular diseases. In a study conducted on 670 patients with T2DM (mean DM duration, 5.1±3.2 years), Guo et al. [8] found perirenal fat tissue to be significantly higher in the group with subclinical atherosclerosis than in the group without it. They demonstrated that perirenal fat tissue was associated with subclinical atherosclerosis and CIMT and concluded that it is an important marker in obesity. In the present study, we also found that PPRFT is related to CIMT, as well as to myocardial functions. Unlike the study by Guo et al. [8], our study group included patients with ND-T2DM. We also measured PPRFT using ultrasound, which is a noninvasive method.

Xu et al. [19] investigated metabolic risk parameters of obesity indicators in 337 patients with T2DM. In addition to anthropometric parameters, such as BMI, waist circumference, and waist/hip ratio, the relationships between metabolic parameters and visceral adipose tissue, subcutaneous adipose tissue, and perirenal fat tissue were examined. The authors reported a significant relationship between perirenal fat tissue and HDL-C, triglyceride, and uric acid levels. In our study, PPRFT values were also associated with glucose, triglyceride, HDL-C, and HOMA-IR levels.

In another study, the relationship between PPRFT and 24-hour blood pressure was examined in 42 individuals who were obese or overweight and did not receive any medication. The results of that study showed that PPRFT was related to 24-hour average diastolic blood pressure. The authors also suggested that PPRFT had a direct effect on blood pressure elevation. In our study, we found that PPRFT values correlated with resting systolic and diastolic blood pressures in patients with ND-T2DM [20].

Similar to the methodology in our study, Lamacchia et al. [21] examined the relationship between PPRFT measured using ultrasonography and the glomerular filtration rate, renal resistance index, and hyperuricemia in 151 patients with T2DM. The authors showed that PPRFT was an independent predictor of renal failure in these patients. The relationship between PAT thickness measured using tomography (and visceral and subcutaneous adipose tissue) and the development of fatty liver disease associated with metabolic dysfunction in patients with T2DM was examined in another study conducted in China. The authors concluded that PAT thickness was an independent predictor of fatty liver disease associated with metabolic dysfunction [22]. Koo et al. [23] investigated the presence of atherosclerosis in six different vascular beds with perirenal fat tissue measured on computed tomography scans of 3919 asymptomatic individuals. They concluded that PAT was associated with atherosclerosis in the renal artery and abdominal aorta. Using a methodology similar to that of our study, Okeahialam et al. [24] evaluated the relationship between perirenal fat tissue and subclinical atherosclerosis in 221 individuals without diagnosed disease and determined whether perirenal fat tissue could be used in cardiovascular risk assessment. The authors found that perirenal fat tissue was associated with CIMT and anthropometric measurements and concluded that perirenal fat tissue is associated with parameters that predict atherosclerosis. In contrast, we found that PPRFT was associated with CIMT in patients with ND-T2DM.

Increased epicardial adipose tissue (EAT) can induce local inflammation, which negatively affects the myocardium and coronary arteries. EAT is a reliable and easily measurable marker for various cardiovascular outcomes [25]. Iacobellis et al. [26] examined whether EAT was associated with anthropometric and clinical parameters of metabolic syndrome in 72 patients with various BMIs. As a result of that research, the authors concluded that there was a significant relationship between EAT detected using echocardiography and visceral adipose tissue, and that waist circumference was the strongest parameter associated with EAT.

In our study, we also found a significant correlation between PPRFT and EAT, and a significant relationship between EAT and CIMT. This observation indicates that EAT, an important preatherosclerotic inflammatory adipose tissue, can predict subclinical atherosclerosis and that PPRFT is related to EAT.

According to our study, PPRFT increased significantly in individuals with ND-T2DM compared to the control group. Subclinical atherosclerosis and deterioration of diastolic function have been detected in individuals with ND-T2DM. In addition, epicardial fat tissue thickness, which is believed to be related to atherosclerosis, was also related to PPRFT. These results are consistent with those of previous studies. The relationship between PPRFT measured at the time of new diagnosis and subclinical atherosclerosis, EF, and diastolic dysfunction in patients with T2DM was found to be statistically significant. Increased PPRFT values may be useful in predicting an increased risk of subclinical atherosclerosis and diastolic dysfunction. The fact that PPRFT is detected noninvasively using ultrasound without radiation exposure makes our study important. Therefore, we believe that PPRFT measured at the time of diagnosis in patients with DM may provide useful information for predicting the risk of atherosclerotic cardiovascular disease and myocardial dysfunction. We believe that the results obtained in our study should be carried forward and the data in the literature further supported by new studies conducted on a larger scale and in different populations.

Our study was primarily a cross-sectional study, and individuals who met the inclusion criteria were included over a certain period when identified. Therefore, the study results may not reflect the population as a whole. Generally, PPRFT shapes, which are markers of visceral fat tissue, vary among individuals and are not defined geometrically. Because of the 2D ultrasonography, variations in the three-dimensional shape of the tissue could have impacted the estimation of thickness. To minimize these differences, we standardized our ultrasonographic measurements, measuring the maximum lengths in the same position and at the same measurements as much as possible. Measurement of fat tissue thickness using magnetic resonance imaging may be an alternative, but it is not compatible with clinical practice in our study group. As the ND-T2DM group included individuals not taking antihypertensive and lipid-lowering drugs and with no history of coronary artery disease, it may not represent the entire population of patients with DM. PAT contains a mixture of brown and white adipocytes. The medial region of the PAT is located around the renal hilum, and several studies have identified brown adipocytes there. In contrast, the lateral region of the PAT predominantly consists of white adipocytes [27]. Epicardial fat typically comprises white adipose tissue but also displays brown-fat-like or beige-fat features [28]. Based on their morphological and functional differences, this must be considered when interpreting our results.

## Figures and Tables

**Table 1.** Comparison of demographic, laboratory, and ultrasonographic parameters between the groups

Characteristic	Group 1	Group 2	*p*-value
No. of patients	111	57	
Age (yr)	49.7±9.6	48.5±7.2	0.391
Female sex	52 (46.8)	34 (59.6)	0.117
Smoking	44 (39.6)	22 (38.6)	0.896
Body mass index (kg/m^2^)	29.4±4.4	25.9±3.7	<0.001
Waist circumference (cm)	103.6±11.2	92.5±11.9	<0.001
Hip circumference (cm)	106.3±10.1	101.0±7.7	0.001
SBP (mmHg)	137.4±20.0	129.9±16.2	<0.016
DBP (mmHg)	85.5±12.4	82.1±12.5	<0.091
Heart rate (beat/min)	85.6±12.9	78.2±8.9	<0.001
Glucose (mg/dL)	198.9± 78.5	93.0±7.2	<0.001
Urea (mg/dL)	27.2±7.5	26.0±7.3	0.313
Creatinine (mg/dL)	0.73±0.18	0.72±0.15	0.715
GFR (mL/min/1.73 m^2^)	101.9±12.7	103.7±9.4	0.155
AST (U/L)	21.9±13.3	18.3±7.9	0.061
ALT (U/L)	30.9±30.3	17.2±10.5	0.001
Total cholesterol (mg/dL)	205.9± 48.9	193.6±33.1	0.096
HDL-C (mg/dL)	44.8±13.2	55.4±11.5	<0.001
LDL-C (mg/dL)	126.3±41.7	116.1±28.6	0.105
Triglyceride (mg/dL)	191.9 ±121.7	107.7±49.9	<0.001
HbA1c (%)	8.85±2.38	5.41±0.30	<0.001
Insulin (mU/L)	13.99±9.11	7.25±3.63	0.002
HOMA-IR	6.10±3.81	1.88±1.22	0.001
C-reactive protein (mg/L)	7.5± 7.8	2.4±3.4	<0.001
Microalbumin	31.7±61.4	30.6±40.1	0.998
Uric acid	5.91±11.3	6.96±15.7	0.745
White blood cell (×10^9^/L)	7.73±2.14	6.85±1.99	0.011
Carotid IMT (mm)	1.14±0.25	0.69±0.12	<0.001
Epicardial FT (mm)	7.20±1.9	4.2±0.8	<0.001
Right perirenal FT (mm)	12.4±4.1	6.5±2.9	<0.001
Right peri+pararenal FT (mm)	24.7±7.5	13.2±5.2	<0.001
Left perirenal FT (mm)	11.5±3.6	7.0±2.8	<0.001
Left peri+pararenal FT (mm)	23.7±6.1	13.9±5.7	<0.001
Mean right and left peri+pararenal FT (mm)	24.2±6.1	13.4±5.1	<0.001

Values are presented as number only, mean±standard deviation, or number (%). SBP, systolic blood pressure; DBP, diastolic blood pressure; GFR, glomerular filtration rate; AST, aspartate transaminase; ALT, alanine transaminase; LDL-C, low-density lipoprotein; HDL-C, high-density lipoprotein; HbA1c, glycated hemoglobin; GGT, gamma-glutamyltransferase; Homa-IR, homeostatic model assessment-insulin resistance; IMT, intima-media thickness; FT, fat thickness.

**Table 2.** Comparison of conventional echocardiography parameters, diastolic and systolic functions, myocardial velocities in newly diagnosed type 2 diabetes mellitus patients and control groups

Parameter	Group 1 (n=111)	Group 2 (n=57)	*p*-value
EF (%)	62.4±2.3	68.3±2.0	<0.001
LVESD (cm)	2.55±0.46	2.45±0.32	0,136
LVEDD (cm)	4.55±0.42	4.63±0.41	0.247
IVS (cm)	1.11±0.20	0.83±0.16	<0.001
PW (cm)	1.11±0.38	0.85±0.15	<0.001
Aortic diameter (cm)	2.72±0.32	2.58±0.31	0.008
Left atrium diameter (cm)	3.52±0.40	3.20±0.41	<0.001
Mitral E (m/sec)	0.63±0.14	0.78±0.16	<0.001
Mitral A (m/sec)	0.77±0.14	0.59±0.13	<0.001
Mitral EDT (ms)	238.3±55.9	200.9±44.7	<0.001
Lateral S′ wave (cm/sec)	9.74±2.14	10.40±2.10	0.860
Lateral E′ wave (cm/sec)	9.14±2.77	12.7±2.61	<0.001
Lateral A′ wave (cm/sec)	10.30±2.61	8.77±2.20	0.001
Septal S′ wave (cm/sec)	7.68±1.34	8.66±1.39	<0.001
Septal E′ wave (cm/sec)	6.49±1.72	9.87±1.89	<0.001
Septal A′ wave (cm/sec)	9.01±1.76	7.37±1.60	<0.001
Tricuspid S′ wave (cm/sec)	12.00±2.32	12.10±2.39	0.883
Tricuspid E′ wave (cm/sec)	8.53±2.21	13.31±2.90	<0.001
Tricuspid A' wave (cm/sec)	13.29±2.89	9.68±2.14	<0.001
E/A	0.85±0.28	1.33±0.23	<0.001
E/E′ lateral	7.43±2.27	6.36±1.76	0.002
E/E′ septal	10.24±2.47	8.10±1.84	<0.001

Values are presented as mean±standard deviation. EF, ejection fraction; LVESD, left ventricular end systolic diameter; LVEDD, left ventricular end diastolic diameter; IVS, interventricular septum; PW, posterior wall; EDT, e wave deceleration time; S′, systolic myocardial rate; E′, early diastolic forward flow velocity; A′, late diastolic forward flow velocity; E/A, mitral diastolic e and a wave ratio.

**Table 3.** Spearman correlation of mean perirenal and pararenal fat tissue thicknesses with several parameters

Parameter	Mean perirenal		Mean pararenal	
	Rho	*p*-value	Rho	*p*-value
Carotid IMT (cm)	0.490	<0.001	0.517	<0.001
Pericardial fat thickness (cm)	0.588	<0.001	0.574	<0.001
Body mass index (kg/m^2^)	0.493	<0.001	0.477	<0.001
Waist circumference (cm)	0.473	<0.001	0.509	<0.001
Hip circumference (cm)	0.327	<0.001	0.354	<0.001
HbA1c (%)	0.521	<0.001	0.602	<0.001
HOMA-IR	0.321	0.001	0.392	<0.001
E/A	-0.512	<0.001	-0.465	<0.001
E/E' septal	0.279	<0.001	0.311	<0.001
E/E' lateral	0.133	0.095	0.180	<0.001

IMT, intima-media thickness; HbA1c, glycated hemoglobin; HOMA-IR, homeostatic model assessment–insulin resistance; E/A, mitral diastolic e and a wave ratio; E′, early diastolic forward flow velocity.

**Table 4.** Univariate and multivariate regression analysis predicting the presence of new onset type 2 diabetes mellitus

Variable	Univariate		Multivariate	
	OR (95% CI)	*p*-value	OR (95% CI	*p*-value
Mean pararenal	1.590 (1.369–1.848)	<0.001	1.296 (1.088–1.543)	0.004
Mean perirenal	1.668 (1.420–1.960)	<0.001	1.355 (1.117–1.644)	0.002
Body mass index	1.234 (1.123–1.357)	<0.001		
Waist circumference	1.087 (1.050–1.124)	<0.001		
Triglyceride	1.017 (1.009–1.024)	<0.001	1.013 (1.088–1.543)	0.004
HDL-C	0.939 (0.913–0.966)	<0.001		

OR, odds ratio; CI, confidence interval; HDL-C, high-density lipoprotein.
